# A phase 1 randomized, double-blind, placebo-controlled, crossover trial of DAS181 (Fludase®) in adult subjects with well-controlled asthma

**DOI:** 10.1186/s12879-016-1358-9

**Published:** 2016-02-01

**Authors:** Rhonda E. Colombo, Charles Fiorentino, Lori E. Dodd, Sally Hunsberger, Carissa Haney, Kevin Barrett, Linda Nabha, Richard T. Davey Jr., Kenneth N. Olivier

**Affiliations:** 1 Division of Infectious Diseases, Georgia Regents University, Augusta, GA USA; 2 Division of Clinical Research, National Institute of Allergy and Infectious Diseases, Bethesda, MD USA; 3 Biostatistics Research Branch, National Institute of Allergy and Infectious Diseases, Bethesda, MD USA; 4 Laboratory of Clinical Infectious Diseases, National Institute of Allergy and Infectious Diseases, Bethesda, MD USA; 5 Division of Infectious Diseases and Travel Medicine, Georgetown University Hospital, Washington, DC, USA; 6 Laboratory of Immunoregulation, National Institute of Allergy and Infectious Diseases, Bethesda, MD USA; 7 Cardiovascular and Pulmonary Branch, National Heart, Lung, and Blood Institute, Bethesda, MD USA

**Keywords:** Influenza, Asthma, Antiviral, DAS181

## Abstract

**Background:**

Influenza virus (IFV) infection is associated with increased morbidity and mortality in people with underlying lung disease. Treatment options for IFV are currently limited and antiviral resistance is a growing concern. DAS181, an inhaled antiviral with a unique mechanism of action, has shown promise in early clinical trials involving generally healthy human subjects. This study was undertaken to assess the safety and tolerability of DAS181 in individuals with underlying reactive airway disease.

**Methods:**

This was a randomized, double-blind, placebo-controlled, crossover phase 1 study of DAS181-F02. Dry particle inhaler administration of 10 mg was done on 3 consecutive days in ten adult volunteers with well-controlled asthma. The primary outcome was the frequency of adverse events (AEs), grade 1 or higher that occurred during each study period.

**Results:**

There were 280 AEs among ten evaluable subjects (56.8 % active; 43.2 % placebo); 90.7 % were grade 1. No grade 3 or higher AEs occurred. A statistically significant association between exposure to DAS181 and experiencing any AE, a grade 1 AE, or a grade 2 AE was not detected. Overall, the majority of AEs were classified as possibly related (35.7 %), unlikely related (38.9 %), or unrelated (15.4 %) to study drug administration. However, there was a statistically significant association between exposure to DAS181 and experiencing a definitely or probably related AE. Respiratory effects, including dyspnea, dry cough, and chest discomfort related to respirations, accounted for all of the definitely related AEs and one of the most common probably related AEs.

**Conclusions:**

DAS181 was safe in this small study of otherwise healthy subjects with well-controlled asthma. However, the generalizability of these results is limited by the small sample size and generally mild nature of the subjects’ asthma at baseline. The increased association of respiratory events classified as probably or definitely related to DAS181 administration suggests caution may need to be employed when administering DAS181 to individuals with less stable reactive airway disease. Further investigation in a controlled setting of the safety and efficacy of DAS181 in a larger population of asthmatic subjects with varying disease activity is warranted.

**Trial Registration:**

ClinicalTrials.gov NCT01113034 *Trial Registration Date:* April 27, 2010

**Electronic supplementary material:**

The online version of this article (doi:10.1186/s12879-016-1358-9) contains supplementary material, which is available to authorized users.

## Background

In 2009, a novel subtype of influenza A virus (IFV A) capable of infecting humans emerged. This virus, designated A(H1N1)pdm09, was responsible for a worldwide pandemic that ultimately caused an estimated 61 million infections and at least 12,700 deaths [1]. Children and young adults were affected in the majority of cases, typically with acute, self-limited illness. Severe infections occurred with higher frequency among specific populations, including individuals who had an underlying medical condition such as a chronic lung disease [2]. Between 24 and 50 % of individuals hospitalized with A(H1N1)pdm09 infection had a history of asthma, and 36 % of adults had a history of chronic obstructive pulmonary disease (COPD) [2].

The 2009 pandemic heightened awareness of the current limitations for treating influenza and brought into stark relief the need for alternative approaches to treating influenza virus (IFV). Similar to the prior seasonal H1N1 subtype virus in circulation, the A(H1N1)pdm09 virus was almost uniformly resistant to the adamantane antivirals. Fortunately, the majority of tested strains retained in vitro susceptibility to the neuraminidase inhibitors oseltamivir and zanamivir, although more than 300 cases of oseltamivir resistant influenza cases were ultimately reported [3]. Treatment options are very limited in suspected cases of oseltamivir resistance particularly in people with underlying lung disease. Until the FDA approved peramivir for influenza treatment in December 2014, the only other licensed antiviral available for treating adamantane and oseltamivir-resistant virus was inhaled zanamivir, an agent that is relatively contraindicated in individuals with pre-existing reactive airway disease due to the potential for bronchospasm [4].

DAS181, a recombinant fusion protein composed of a sialidase catalytic domain and a cationic amphiregulin (AR) glycosaminoglycan-binding sequence, offers a novel approach to the treatment of IFV [5]. DAS181 works by targeting the host cell receptors to prevent IFV attachment and spread. The host cell receptors for IFV A and IFV B viruses are cell surface sialic acids. The sialidase catalytic domain in DAS181 selectively cleaves sialic acids from the host cells, thereby rendering them refractory to binding by virus particles that require sialic acid as receptors. By binding to the negatively charged glycosaminoglycans on the surface of airway epithelial cells, the cationic C-terminal AR tag anchors DAS181 on the respiratory epithelium, thereby improving treatment potency and retention of the drug on the airway surface [5]. The unique mechanism of action potentially affords DAS181 a broad spectrum of antiviral activity against any virus that uses host cell surface sialic acids as receptors, including all strains of IFV A and IFV B.

In previous studies enrolling healthy subjects, DAS181 has had an acceptable safety profile with no reported serious adverse events, no significant alterations in lung function, and no anaphylaxis or hypersensitivity reactions in phase I studies. DAS181 also showed promising antiviral activity during a phase II study in IFV positive but otherwise healthy adult subjects [6]. DAS181 was well tolerated in this phase II trial, with only 2 (2 %) serious adverse events (AEs) in both the DAS181 treated and placebo group. There were more mild to moderate treatment-related, treatment-emergent adverse events in the DAS181 treatment group than the placebo, with the most common being a transient elevation in serum alkaline phosphatase. There was one death, due to pneumonia, in the DAS181 group, however this was assessed as not related to the study drug and occurred in a patient with previously unknown HIV infection. Inasmuch as the clinical trials to date have focused on subjects without known chronic health conditions, there is a strong need to investigate this agent in subjects with underlying lung disease as well. These individuals are at increased risk of developing influenza-related complications yet have fewer available treatment options given the relative contraindication to use of zanamivir in patients with underlying reactive airway disease. DAS181, like zanamivir, is administered as a dry particle inhalant. However, in phase I and phase II studies to date, there has been no evidence of bronchial hyperreactivity among healthy subjects receiving DAS181.

This research study was undertaken to evaluate the safety and tolerability of DAS181 (Formulation DAS181-02) in individuals with well-controlled asthma.

## Methods

### Study design

**Fig. 1:**
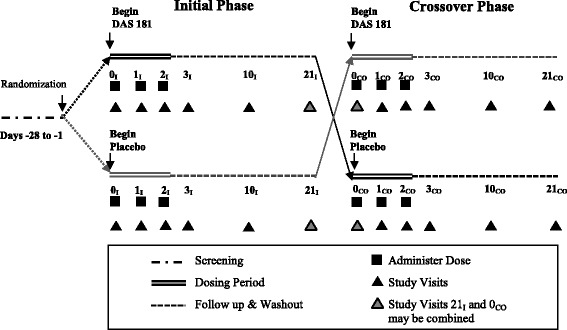
Schematic of study design. Subjects who met eligibility criteria during screening were randomized to receive three consecutive daily doses of either DAS 181 or placebo starting on day 0, the first day of the initial period. Subjects were evaluated at specified time-points for an additional 18 days, then crossed-over to the other treatment group within 6 weeks of completing the initial period. Abbreviations: I = initial; CO = crossover

This was a randomized, double-blind, crossover phase 1 study to evaluate the safety and tolerability of three sequential doses of DAS181 10 mg in adult subjects with well-controlled asthma (Fig. 1).

### Study population

Adult subjects with documented asthma were recruited from the local community. Asthmatic volunteers who had been on stable medication regimens for at least 3 months, were not taking oral corticosteroids or theophylline, and did not have any other significant medical problems were eligible to undergo screening. Following written informed consent, subjects who met entry criteria (Additional file 1: Tables S1A and S1B) were enrolled in the study. The planned study enrollment size of 12 subjects with asthma (10 evaluable plus 20 % drop-out) was chosen to obtain a preliminary assessment of safety and tolerability, while minimizing potential risks to human subjects. The study was stopped once ten evaluable patients completed both arms of the study.

### Study agents

The DAS181-F02 formulation tested in this trial was a dry powder composed of microparticles designed to deposit in the upper airways. The placebo was a similarly appearing powder comprised of lactose monohydrate. DAS181 dry powder or placebo was packaged into clear capsules and delivered using a dry powder inhaler (Cyclohaler®). All study subjects received training in appropriate use of the Cyclohaler® prior to dosing.

### Study interventions

Eligible subjects enrolled in the study were randomized to initially receive either 10 mg of DAS181-F02 or matched placebo via dry particle inhalation on 3 consecutive days. Each subject was followed for an additional 18 days then crossed over to the other treatment group (Fig. 1). The first crossover visit could occur on the same day as the initial period final visit or up to 6 weeks later. Data were collected at specified time points (Additional file 1: Table S1C: Schedule of Events/Procedures).

Subjects were blinded to their allocation and were dosed with the study drug or placebo in a clinical research ward at the National Institutes of Health (NIH) Clinical Center. The subjects were eligible to receive the study drug or placebo on either an outpatient or inpatient basis. All subjects were monitored for at least 1 h following drug administration. If a study subject experienced an acute change in respiratory status, i.e., >10 % drop in the forced expiratory volume in 1 s (FEV1) or a drop in oxyhemoglobin saturation to <90 %, monitoring was extended until the subject’s status returned to near baseline.

### Objectives and outcomes

#### Primary

The primary objective of this study was to evaluate whether DAS181-F02 was safe in individuals with well-controlled asthma. The primary outcome was the frequency of adverse events, grade 1 or higher that occurred during each study period (active drug vs. placebo).

#### Secondary

Additional data was collected to aid in the development of future efficacy studies. Data were collected on standard measures of lung function, airway reactivity, acute exacerbations of asthma, the need for rescue medications, and/or changes in perceived health related quality of life. The specific exploratory outcomes assessed are contained in Additional file 1: Table S1D.

### Study evaluations

Safety and tolerability assessments conducted throughout the study period included assessment of adverse events based on medical history, physical exam findings, vital sign measurements, clinical laboratory test results, chest radiograph, spirometric lung function test, 6 min walk test, and electrocardiography. Sputum evaluation was also performed.

With regard to lung function, acute tolerability of DAS181 was assessed by changes in FEV1 and oxyhemoglobin saturation before and after each dose. Chronic effects on lung function were assessed by change in FEV1% over time, comparing pre-dose measures at baseline (Day0) to measures on the day following sequential dosing (Day 3). Change in exertional capability was evaluated using a standard 6-minute walk assessment at baseline and day 3 [7]. Asthma exacerbation was a clinical diagnosis, defined according to national guidelines [8] as some combination of worsening shortness of breath, cough, wheezing, and/or chest tightness.

Methacholine challenge testing to assess airway reactivity was performed according to a standardized protocol [9]. Subjects inhaled a nebulized mist prepared from a series of increasing concentrations of methacholine. FEV1 was computed after each concentration was administered, and the concentration of methacholine required to produce a decrease of 20 % in FEV1 was recorded as the provocative concentration producing a 20 % decline (PC_20_). Change in airway reactivity was measured by the relative change in PC_20_ from screening to Day 3.

Sputum was induced using a nebulized 3 % sodium chloride solution according to standard procedure. The induced sputum was processed for semi-quantitative Gram’s stain and bacterial culture. Quantitative assessment of the immunologic cells present in the induced sputum was planned, however was ultimately not performed due to inadequate sample collection.

Blood samples were collected at specified time points for safety evaluation. In the case of abnormal activated partial thromboplastin time (aPTT) values, further investigation with a Kaolin clotting time (KCT) was performed to evaluate whether there was a true coagulation abnormality or an in vitro phenomenon related to the sialidase potentially interfering with in vitro assays via phospholipid interactions.

Changes in perceived health related quality of life at day 10 versus baseline were assessed using three questionnaires validated for use in asthma: the acute Short Form-36 (SF-36) Health Survey, the Asthma Control Survey (ACS), and the Asthma Quality of Life Questionnaire (AQLQ). The acute SF-36 is a generic self-administered health questionnaire comprised of 36 questions that yield an 8-scale profile of functional health and well-being with a 1-week recall period [10]. The ACS has 7 question designed to measure asthma control and change in asthma control; use of a shortened version that excludes measurement of either FEV1 or peak flow has also been validated [11]. The AQLQ is a 32-question questionnaire developed to measure the primary functional problems in adults with asthma and encompasses 4 domains: symptoms, activity limitation, emotional function, and environmental stimuli [12].

### Study oversight

This study was approved by the National Institutes of Allergy and Infectious Diseases (NIAID) Institutional Review Board and was conducted in compliance with Good Clinical Practice Guidelines, the Declaration of Helsinki, and local regulatory requirements. The study was designed and conducted by NIAID investigators at the NIH and was funded by the Division of Intramural Research. The study drug, delivery device, and matched placebo were provided by the manufacturer of DAS181, Ansun Biopharma, Inc. The study agent and placebo were pre-randomized by the NIH Pharmaceutical Development Section (PDS) using a computer generated sequence. The randomization code was maintained by the PDS in a sealed envelope and stored in a locked cabinet with access available only for emergencies. Block randomization was done for the first four patients followed by review of safety data by an independent Safety Monitoring Committee. Stopping rules included any serious adverse event or Grade 4 adverse event deemed definitely or probably related to the study drug, anaphylactic or other life threatening reaction or acute drop in FEV1 to <50 % in response to study drug administration, or observed hemolysis associated or probably associated with the study drug.

### Analysis

#### Primary endpoint

To assess safety, the percentage experiencing at least one severe adverse event (SAE) was estimated by treatment arm, along with 95 % confidence intervals (CIs). The number and percentage of participants who experienced at least one AE of any severity and 95 % CIs were also determined. Additionally, AE rates by type of AE were tabulated by severity and relationship to treatment.

Each adverse event was graded according to the 2009 Division of AIDS (DAIDS) Table for Grading Severity of Adverse Events [13]. For all collected AEs, the blinded clinician who examined and evaluated the subject determined the AE’s causality based on temporal relationship and his/her clinical judgment. The degree of certainty about causality was graded as definitely related, probably related, possibly related, unlikely related, unrelated, or as an expected event related to disease process. The study’s principle investigator (PI) made the final determination of AE grading and study drug relatedness prior to unblinding.

AEs were recorded for all randomized subjects who received at least one dose of study drug or placebo. However, only those subjects who completed both periods of the study were considered evaluable for safety analysis due to the pre-specified constraints of the crossover design. Events that occurred prior to screening and those that occurred after screening but prior to the first dose of initial period study drug (active or placebo) were considered pre-existing and were thus not included in the analysis.

#### Secondary endpoints

As a secondary analysis, the AE rates by period were examined. Analyses included the estimation of 1) the probability of an AE during the control period but no AE during the active drug period, and 2) the probability of no AE during the control period but an AE during the active drug period. The difference in AE rates by treatment arm was assessed using a McNemar’s test with continuity correction. With ten subjects this test had 80 % power if the true rate of patients having an AE with control was 0 and with treatment was 0.65.

For categorical variables, the numbers and percentages were tabulated; for continuous variables, the mean and standard deviation (SD) were determined for normally distributed values and median and interquartile range (IQR) were determined for variables with non-parametric distribution. For continuous variables, paired t-tests and nonparametric methods, such as the Wilcoxon signed rank test, were used to assess differences in the patient experience on active versus placebo. Statistical analysis was conducted using Excel and SAS Enterprise Guide version 4.3 (SAS Inc., Cary, NC 2006–2010).

## Results

### Demographics

A total of 22 subjects were screened to enroll 11 subjects (50 % screen failure rate). Among these 11 subjects who were randomized to receive study drug, one subject voluntarily withdrew from the study after completing only the initial period due to scheduling conflicts. As he did not complete both periods he was excluded from further analysis; after completion of the study it was determined that he had received placebo. Table 1 contains the demographic characteristics for the ten evaluable subjects. Each subject served as his or her own control for this crossover study.

**Table 1:** Demographics and Baseline Characteristics of Study Subjects

Characteristic	Evaluable subjects
	*N* = 10
Age (years)	
Median (IQR)	22.5 (22, 40)
Sex	
Female	7 (70 %)
Male	3 (30 %)
Race	
White	5 (50 %)
Black or African American	3 (30 %)
Asian	1 (10 %)
Multi-Racial	1 (10 %)
Body Mass Index (kg/m^2^)	
Median (IQR)	23.0 (21.4, 24.3)
FEV1, Baseline (day 0)	
Median (IQR)	3.09 (2.78, 3.62)
FEV1 % Predicted, Baseline (day 0)	
Mean (SD)	95.1 (11.6)
PC_20_, Baseline (screening, mg/m^2^)	
Median (IQR)	1.79 (0.49, 3.62)
Asthma Medications (baseline)	
Short Acting Beta Agonist, inhaled	9 (90 %)
Long Acting Beta Agonist/Glucocorticoid, inhaled	2 (20 %)
Glucocorticoid, inhaled	1 (10 %)
Leukotriene-receptor antagonist	2 (20 %)

Abbreviations: *IQR* interquartile range, *FEV1* forced expiratory volume in 1 second, *SD* standard deviation, *PC*_*20*_ provocative concentration producing a 20 % decline (PC_20_)

### Primary outcome: adverse events

**Fig. 2:**
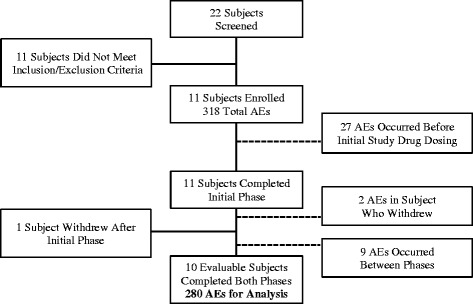
Flow chart depicting reasons for exclusion of subjects and adverse events from final analysis. Half of the 22 screened subjects failed to meet inclusion or exclusion criteria. One enrolled subject withdrew after the initial period thus his data was excluded from the final analysis according to protocol. Adverse events that occurred before dosing or between periods were also excluded. Ultimately, ten subjects accounted for 280 adverse events in the final analysis. Abbreviations: AEs = adverse events

A total of 318 AEs occurred among the 11 enrolled subjects after screening and before study conclusion. However, 29 (9.1 %) were excluded from analysis because they did not fulfill the pre-specified criteria for inclusion as they occurred after screening but before dosing during the initial period and/or occurred in the subject who did not complete both periods of the study. Nine of the remaining AEs were documented between the last study point of the initial period (I/21) and the initial dose of study medication (placebo or active) on day 0 of the crossover period. These were characterized as “between- period” AEs. All 9 had been classified as either unrelated (89 %) or unlikely related (11 %) to the study drug. Analysis was performed with and without inclusion of the between- period events and no appreciable effect on results was noted, thus these events were excluded, resulting in 280 AEs for final analysis. Figure 2 depicts the reasons for exclusion of subjects and AEs from final analysis.

None of the subjects experienced any grade 3 or 4 AEs, thus the main primary endpoint (percentage of subjects experiencing at least one SAE) was 0 % for each treatment arm. All subjects experienced at least one grade 1 AE during each treatment period. The probability of detecting any adverse event during the active period but not during placebo period, and vice versa, was thus 0. Among all grades, 159 (56.8 %) AEs occurred during the active period and 121 (43.2 %) during the placebo period. There were 254 (90.7 %) grade 1 and 26 (9.3 %) grade 2 AEs. Of the grade 1 AEs, 136 (53.5 %) occurred following DAS181 and 118 (46.5 %) occurred following placebo administration. The majority (23; 88.5 %) of grade 2 AEs were observed during the active drug period. Four (40 %) of the subjects experienced a grade 2 AE in the active drug but not the placebo period, whereas only 1 (10 %) of the subjects had a grade 2 AE in the placebo but not the active drug period. However, this association between exposure to DAS181 and grade 2AE was not statistically significant (exact test *p* = 0.375).

**Fig. 3:**
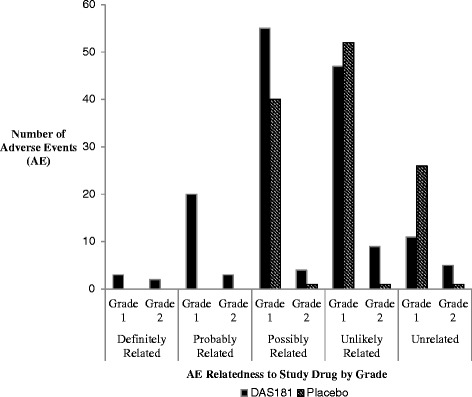
Distribution of adverse events by grade and relatedness, according to study period. Of the 280 total AEs, 56.8 % occurred during the active period and 43.2 % during the placebo period. 254 (90.7 %) AEs were grade 1 and 26 (9.3 %) grade 2. While only 28 (10 %) AEs were classified as definitely or probably related, all 28 occurred during the active period, representing a statistically significant association between exposure to DAS181 and experiencing a definitely or probably related AE (8 discordant pairs, exact test *p* = 0.0039)

With regard to assigned relatedness of study drug administration to AE, there were 5 (1.8 %) AEs classified as definitely related, 23 (8.2 %) probably related, 100 (35.7 %) possibly related, 109 (38.9 %) unlikely related, and 43 (15.4 %) unrelated. Figure 3 graphically depicts the AEs by relatedness to study drug and grade, according to period. Of the 28 definitely or probably related AEs, all occurred during the active drug period of study; this represented a statistically significant association between exposure to DAS181 and experiencing a definitely or probably related AE (8 discordant pairs, exact test *p* = 0.0039).

Overall, AEs most commonly occurred on study days 2 (50/280, 17.9 %), 1 (47/280, 16.8 %), and 3 (43/280, 15.4 %). When examined by period, AEs were most likely to occur on days 2 (21/121, 17.4 %), 3 (20/121, 16.5 %), and 10 (20/121, 16.5 %) during the placebo period, and days 1 (30/159, 18.9 %), 2 (29/159, 18.2 %), and 3 (23/159, 14.5 %) during the active drug period.

**Fig. 4:**
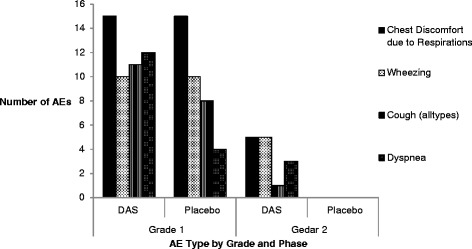
Distribution of most common adverse event types by study period and grade. The respiratory symptoms depicted in this figure accounted for 99 adverse events and represented four of the most common adverse events overall. The variable depicted as “Cough (all types)” included all cough related AEs including dry cough, cough not otherwise specified, and productive cough. The majority of AEs were grade 1, however all grade 2 AEs in this group occurred during the active period

The most common AE overall was chest discomfort related to respiration (35/280, 12.5 %), followed by microscopic hematuria (27/280, 9.6 %), wheezing (25/280, 8.9 %), cough of any type (20/280, 7.1 %) and dyspnea (19/280, 6.8 %). The majority of instances of AE due to microscopic hematuria were attributable to a discrepancy between the normal range for urine red blood cells used by the NIH Department of Laboratory Medicine and the DAIDS standard scale which was employed per protocol for AE grading, rather than a true clinical abnormality. Figure 4 shows the distribution of the most common AEs by period and grade, excluding microscopic hematuria as it was deemed unlikely to be relevant. When assessed by assigned relationship, dyspnea (3/5, 60 %), dry cough (1/5, 20 %), and chest discomfort related to respirations (1/5, 20 %) were the types of AEs classified as definitely related; all definitely related AEs occurred in a single subject. The four most common AEs classified as probably related were chest discomfort related to respirations (4/23, 17.4 %) elevated alkaline phosphatase (4/23, 17.4 %), elevated aspartate aminotransferase (3/23, 13 %), and prolonged aPTT (3/23, 13 %). One subject had a decrease in FEV1 to <80 % predicted during the active drug period that was designated as probably related.

### Specific safety laboratory findings of note

#### Alkaline phosphatase

There were seven grade 1 and one grade 2 elevations in alkaline phosphatase levels among six subjects during the course of the study. All occurred during the active drug period, with peak values recorded at day 3 or day 10. All alkaline phosphatase values had returned to baseline prior to crossover in those subjects who had elevations in the initial period, Interestingly, the grade 2 elevation was detected on day 3 in the same subject in whom the third dose of study drug was withheld due to concerns about adverse respiratory effects.

#### PTT/KCT

Six subjects had minor elevations in aPTT; however, only three had abnormalities that reached grade 1 on the DAIDS severity scale. All grade 1 elevations of aPTT were transient and occurred on day 2 or 3 of the active drug period; all resolved by the day 10 follow up visit. All corresponding KCTs were within normal limits.

#### Microbiology

There were a total of four positive sputum cultures from study subjects while on study. The identified organisms included *Moraxella (Branhamella) catarrhalis*, Beta-hemolytic *Group F Streptococcus*, *Haemophilus influenzae*, and methicillin susceptible *Staphylococcus aureus*. All accompanying gram stains revealed squamous epithelial cells and mixed oropharyngeal flora suggesting oropharyngeal contamination. None of the organisms were felt to be associated with true infection and thus were not treated with antibiotics. Only the *Group F Streptococcus* was isolated during the DAS181 treatment period; the other three were isolated during the placebo period. One additional subject had *Neisseria meningitidis* isolated from sputum 2 months after completing the study; of note, that subject had received DAS181 during the initial period and had multiple sputum samples with negative cultures during both periods.

### Secondary outcomes

#### Change in post-dose FEV1

**Fig. 5:**
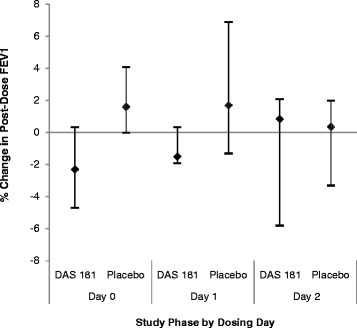
Percent (%) change in post-dose FEV1 compared to pre-dose, by dosing day. The change in median FEV1 following DAS181 versus placebo dosing was not statistically different either overall (*p* = 0.45) or by dosing day

There was a 50 milliliter (mL) [IQR −110 mL, 40 mL] overall decrease in median FEV1 following administration of DAS181, and a 35 mL [−40 mL, 140 mL] increase after placebo dosing, representing a −1.9 % and 1.1 % change, respectively. This overall difference was not statistically significant (*p* = 0.45), nor were any of the differences by dosing day. Of note, one subject did experience a clinically significant decrease in FEV1 after receiving their 3rd dose of DAS181 (430 mL decrease; 11.7 %); the same subject also had a 340 mL decrease (10.1 %) with their first dose of placebo (crossover period) and a 20.8 % increase with the 3rd dose of placebo. Figure 5 shows the median % change in FEV1 after dosing with either DAS181 or placebo, examined by day.

#### Day 3 change in FEV1 from baseline

The mean change from baseline FEV1 % to 24 h after the 3rd dose (Day 3) was −1.7 % (median −2, IQR 11) decrease for DAS181 and - 2.7 % (median 1, IQR 16) decrease for placebo. There was not a statistical difference by treatment period.

#### Acute change in oxyhemoglobin saturation

No subject experienced a significant decline in oxyhemoglobin saturation (SpO2), specified a priori as ≥4 % decline in SpO2, following inhalation of either DAS181 or placebo. The mean acute change (post dose minus pre dose) in SpO2 was 0.2 % ± 1.4 % following DAS181 administration and 0.5 % ± 1.4 % following placebo. The maximum decrease in SpO2 1 h post dose for each period was 3 % with active study drug and 2 % with placebo.

#### Acute asthma exacerbations of underlying lung disease

Two subjects (20 %) experienced grade 2 events characterized as asthma exacerbations during the study period. Both exacerbations occurred on day 0 of the active period following administration of the initial dose of DAS181. The final dose of DAS181 was withheld at the investigators’ discretion from one of the subjects who experienced an asthma exacerbation during the study period. None of the subjects had documented asthma exacerbations during the placebo period however one additional subject did experience an asthma exacerbation after completing screening but before receiving a dose of either DAS181 or placebo.

#### Daily peak expiratory flow readings

The morning peak flow was similar in each study period, with an overall median of 449 L/min [IQR 342, 532] in the DAS181 period and 429 liters per minute (L/min) [IQR 339, 546] in the placebo period. However, half the subjects had missing peak flow readings thus limiting the validity of this measurement and further analysis.

#### Airway hyper-responsiveness

There was no appreciable adverse effect on airway hyper-responsiveness with DAS181 24-hours after dosing completed (*p* = 0.1093).

#### 6-minute walk distance and oxygen saturation

There was a small increase in median distance walked on day 3 compared to baseline in both periods (DAS181: 19 m [IQR −9, 43]; placebo: 11.5 m [−14, 42]). None of the subjects experienced a clinically significant decline in SpO2 on either period. The median change in Sp02 with exertion was 0 % [IQR 0, 1] in the active period and a decline of 1 % [IQR −1, 1] in the placebo period.

#### Rescue medication usage during active drug or placebo study period

Use of rescue medication was documented a total of 77 times during the study, 58 times in active drug period [median 0, range 0 to 9] and 19 times in placebo period [median 0, range 0 to 2]. During the active period, the highest uses of rescue medication occurred on dosing Days 0 (10), 1 (10), and 2 (12). There was not a clearly discernible pattern during the placebo period. Although there was a significant difference in the total number of times rescue medication use was recorded on active versus placebo period (paired *t*-test, *p* value 0.008), there was not a significant association between exposure to DAS181 and the need for any rescue medication use (McNemar’s statistic, *p* = 0.371). This may be related to one subject accounting for much of the difference in total medication usage, recording rescue medication use 20 times in the active period and 2 in the placebo.

#### Health related quality of life

Health related quality of life was assessed via multiple modalities. There were not any detectable differences by period on SF-36, nor did any of the mean change values reach a level considered to be a minimally important difference (MID). Similarly, neither a statistically significant nor a MID from baseline was detected between periods with the ACS. There was a statistically significant difference in the overall mean change in AQLQ from baseline (*p* = 0.0055), however the mean difference (−0.175) did not reach the MID standard for AQLQ (0.5).

## Discussion

Influenza virus infection is a major cause of morbidity and mortality worldwide. There are limited treatment options for IFV currently available, and emerging viral resistance threatens existing agents. Individuals with underlying lung disease have historically been among the highest risk for IFV related complications; however, they may also be more prone to treatment related adverse effects, most notably respiratory problems, particularly when the agent is administered by inhalation.

Prior phase I and II studies of DAS181, an inhaled antiviral with a unique mechanism of action that may be associated with a higher barrier to antiviral resistance development, have been promising in human subjects who have been generally healthy at baseline. Among subjects without known underlying disease, there have not been any significant alteration in lung function detected, nor has there been a significant difference in the rate of serious adverse events with DAS181 treatment versus placebo [6]. DAS181 has also showed promising antiviral activity, suggesting that further development of this novel antiviral drug is warranted. Assessing the safety of this inhalational agent in individuals who have underlying comorbidities, particularly lung disease, is necessary to help guide such development.

In this small, phase I study, DAS 181 was not associated with any serious adverse events (grade 3 or higher) in well-controlled subjects with asthma. There were more total AEs in the DAS181 period than the placebo (56.8 % vs. 43.2 %), and 88.5 % of the grade 2 AEs occurred during the active period. However, a statistically significant association between exposure to DAS181 and experiencing any AE, a grade 1 AE, or a grade 2 AE was not detected.

With regard to the respiratory effects of DAS181 in subjects with asthma, there were mixed findings. On the one hand, there were no clinically significant changes in oxyhemoglobin saturation or detectable worsening of airway hyper-responsiveness with DAS181 administration. There also was not a significant overall change in airflow, measured by post-dose FEV1, in the active vs placebo period. On the other hand, one subject did experience a significant (>10 %) acute decline in FEV 1 after DAS181 administration, but also experienced a similar decline after inhalation of placebo. Another subject’s percent predicted FEV1 dropped below 80 % acutely following DAS181 dosing. There was also a statistically significant association between exposure to DAS181 and experiencing a definite or probably related AE of any grade, with respiratory complaints being the most common of these AEs. Two subjects experienced events characterized as an asthma attack after the initial dose of DAS181 was administered; the final dose of DAS181 was not administered to one of these subjects due to respiratory concerns. Both subjects who experienced asthma exacerbations were successfully managed with an inhaled corticosteroid.

This study was undertaken to investigate the safety and tolerability of DAS181 in subjects with well controlled asthma. The crossover design allowed subjects to serve as their own controls, thus decreasing one of the most prevalent confounding factors in asthma research, inter-subject variability. The relatively complex study design required by the crossover comparisons also permitted each participant to undergo more frequent clinical assessments than usual, increasing our ability to assess safety and monitor for transient AEs. The relatively small size of the study did influence the power to detect significant differences between groups, thus affecting the strength of formal comparisons. However, for safety reasons, this small number was by design. The goal of ten evaluable subjects with asthma completing both periods was reached, permitting preliminary assessment of safety and tolerability while minimizing the potential risk to human subjects being exposed to an investigational agent.

## Conclusions

In summary, DAS181 was generally well tolerated in this small study of otherwise healthy subjects with mild, well-controlled asthma. However, the small sample size and generally well-controlled nature of the subjects’ asthma at baseline limits generalizability of the result to all asthmatics, particularly those at the severe end of disease activity. Although there were no serious adverse effects detected that would preclude further investigation of this agent in people with underlying asthma, the possibility that individuals who have more reactive airway disease at baseline may experience more adverse effects following inhalation of the study drug remains. The increased association of respiratory events classified as being probably or definitely associated with DAS181 in this study, including asthma exacerbations in two subjects, suggests that caution may need to be employed when administering DAS181 to individuals with less stable reactive airway disease. Further investigation in a controlled setting of the safety and efficacy of DAS181 in a larger population of asthmatic subjects at different levels of disease activity is clearly warranted.

## Additional file

Additional file 1:
**Supplementary tables delineating additional pertinent study information, including inclusion and exclusion criteria, the schedule of study events, and the exploratory outcomes investigated.** (DOCX 39 kb)

## Abbreviations

IFV: influenza virus
AEs: adverse events
IFV A: influenza A virus
COPD: chronic obstructive pulmonary disease
AR: amphiregulin
IFV B: influenza B virus
NIH: National Institutes of Health
FEV1: forced expiratory volume in 1 second
PC_20_: provocative concentration producing a 20 % decline (PC_20_)
aPTT: activated partial thromboplastin time
KCT: kaolin clotting time
ACS: asthma control survey
AQLQ: asthma quality of life questionnaire
NIAID: National Institute of Allergy and Infectious Diseases
SAE: severe adverse event
CI: confidence interval
DAIDS: division of AIDS
PI: principal investigator
SD: standard deviation
IQR: interquartile range
n: number of episodes
mL: milliliter
SpO2: oxyhemoglobin saturation
L/min: liters per minute
MID: minimally important difference
