# Supplementary material for: A phase 1 randomized, double-blind, placebo-controlled, crossover trial of DAS181 (Fludase®) in adult subjects with well-controlled asthma
Source: BMC Infect Dis. 2016 Feb 1;16:54. doi: 10.1186/s12879-016-1358-9 (PMC4736611; doi:10.1186/s12879-016-1358-9)
Supplement: Additional file 1: — Supplementary tables delineating additional pertinent study information, including inclusion and exclusion criteria, the schedule of study events, and the exploratory outcomes investigated. (DOCX 39 kb) [file 12879_2016_1358_MOESM1_ESM.docx]

| **Supplemental Table A. Inclusion Criteria for Subject with Asthma**   1. A male or female subject must be ≥18 to ≤65 years of age. 2. Except for underlying airway disease, subject must be in good health as determined by medical history, targeted physical examination based on medical history, and vital signs (that include temperature, blood pressure, heart rate, and pulse oximetry). 3. Subject must be able to verbalize understanding of the informed consent form, verbalize willingness to complete all study procedures, and provide written informed consent (sign the informed consent form). 4. Subject must be willing to commit to participating in both the initial and cross-over stages of the study. 5. Dipstick analysis of subject’s urine specimen must be negative or show only trace amounts of glucose, hemoglobin, and protein. A menstruating female who tests positive for urine hemoglobin may be retested. 6. Subject must have blood screening test results that are within normal limits (according to standards set within the Clinical Center) for the following tests: alanine transaminase (ALT), alkaline phosphatase (ALKP), activated partial thromboplastin time (APTT), and aspartate transaminase (AST). 7. Subject must have hematologic screening tests that are within a specified range, including hemoglobin of ≥10.9 g/dL, white blood cell count ≥2500/mm^3^, and platelet count ≥125,000/mm^3^ (all < grade 1 on DAIDS severity scale). 8. A female subject must be post-menopausal (≥1 year without menses), have been surgically sterilized, practice abstinence, or use an effective method of birth control that may include an intrauterine device, spermicide, barrier, and hormonal contraception. A female subject must also have a negative serum test for pregnancy during the Screening period, and a negative urine test for pregnancy on each day of drug or placebo administration.   Asthma Subjects:   1. Subject must have a clinically established diagnosis of asthma based upon a history of episodic symptoms of airway obstruction or airway hyper-responsiveness (i.e., wheezing). 2. Subject must have a documented increase in FEV1^i^ or FVC^ii^ ≥12% (and at least 200 mL) from baseline after inhaling a short-acting bronchodilator; or a PC_20_FEV1^iii^ response to methacholine of ≤8 mg/mL. 3. At the time of study enrollment, subject’s asthma has been well-controlled for at least the past 3 months, as defined by the following. ^v^    1. Daytime symptoms occur ≤2 days per week    2. Normal daily activity is not limited by asthma    3. Nocturnal symptoms/nighttime awakenings ≤2 times/month    4. FEV1 ≥80% predicted    5. Use of short-acting beta-agonist ≤2 days/week    6. Exacerbations requiring oral corticosteroids occur ≤2 times/year    7. There has been no change in asthma medication dose or regimen within 3 months of study enrollment    8. Not currently taking oral corticosteroids   ^i^ FEV1 = forced expiratory volume in 1 second  ^ii^ FVC = forced vital capacity  ^iii^ PC20FEV1 response to methacholine = concentration of methacholine that produces a 20% decrease in FEV1 from the post-saline value during the methacholine challenge  ^iv^ NHLBI. National Asthma Education and Prevention Program. Expert Panel Report 3: Guidelines for the Diagnosis and Management of Asthma. 2007. |
| --- |

| **Supplemental Table B: Exclusion Criteria For Study Subjects**   1. Subject has received any investigational drug or vaccine within 4 weeks prior to study drug dosing, or is planning to participate in another investigational drug or vaccine trial prior to completion of this study. 2. Subject is currently taking theophylline or oral corticosteroids. 3. Subject is allergic to milk or milk products. 4. Subject currently smokes tobacco or has smoked tobacco within 1 year prior to study enrollment. 5. Subject has a baseline requirement for oxygen supplementation. 6. Subject is unable to maintain an oxyhemoglobin saturation of ≥90% during and after 6-minute walk test. 7. The subject tests positive for human immunodeficiency virus (HIV), for hepatitis B virus (HBV), or hepatitis C virus (HCV). 8. The subject’s resting blood pressure is outside normal limits (defined as: systolic 90-140 mmHg; diastolic 50-90 mm Hg). 9. The subject’s heart rate is less than 45 or greater than 100 beats per minute at rest. 10. The subject weighs less than 45 kg. 11. The subject has a Body Mass Index of greater than 35 kg/m^2^. 12. The subject has experienced an episode of acute upper respiratory tract infection, pneumonia, otitis, bronchitis, or sinusitis within 6 weeks of study enrollment. 13. The subject has an oral temperature above 37.8°C (100°F). 14. The subject has any surgical, medical, or laboratory condition that, in the judgment of the clinical investigator, might interfere with the safety, distribution, metabolism, or excretion of the drug. 15. The subject has overt primary ciliary dyskinesia, allergic bronchopulmonary aspergillosis, or cystic fibrosis. 16. The subject has previous or current history of the following conditions: renal, hepatic, cardiac, hematologic (including sickle cell disease and any bleeding disorder or history of abnormal bleeding), muscular, neurological, metabolic, or immunological disorders, malignancy, hepatitis or cirrhosis, transplant recipients, HIV-infection, or other immunosuppressive illness, which could, in the opinion of the study investigators, compromise subject safety or interfere with the assessment of study drug safety. 17. A female who is pregnant or breast-feeding. 18. A subject who has received blood products within 6 months of study enrollment. 19. The subject has donated or lost more than 500 mL of blood in the 3 months prior to screening. 20. The subject has clinically significant medical or psychological conditions that would compromise the subject’s safety, influence the results of the study, affect the subject’s ability to participate in the study, or impair the subject’s ability to provide informed consent. 21. The subject has a history of alcoholism, drug dependence, or significant psychiatric illness within 2 years of study enrollment. 22. The subject uses anticoagulant medications or drugs with known potential for hepatotoxicity as such agents could interfere with relevant safety assessments. 23. The subject is currently using or has recently (within 14 days) used aspirin or clopidogrel. |
| --- |

| **Events** | **Visit 1: Screening** | **Visit 2-4 & 7-9:**  **Study Drug Dosing** | **Visit 5 & 10**  **Follow-up** | **Visit 6 & 11**  **Follow-up  & PK** | **Visit 7 & 12**  **Final** |
| --- | --- | --- | --- | --- | --- |
|  | Day (-28 to -1) | Days 0, 1, and 2 | Day 3 | Day 10 (±3) | Day 21 (±3) |
| Informed Consent | X (Screening IC) | X (Study IC signed 1^st^ thing on Day 0_I_) |  |  |  |
| Incl./Excl. Criteria | X | X |  |  |  |
| Medical History / Demographics | X |  |  |  |  |
| Subject I.D. assignment | X |  |  |  |  |
| DAS181/Placebo administration |  | X (Days 0, 1, 2) |  |  |  |
| AE and SAE Assessment | X | X | X | X | X |
| Clinical Evaluations | Vital sign^i^  Physical exam | Vital signs: pre-dosing and 1 hours post dose (Days 0,1,2)  Physical Exam | Vital signs  Physical exam | Vital signs  Physical exam | Vital signs  Physical exam |
| Electrocardiogram (ECG) | X | X (Pre-dosing Day 0) | X |  | X |
| Spirometric Lung Function Test | X | Pre-dosing and 1 hour post dosing (Days 0, 1, 2) | X | X^ii^ | X |
| Chest X-Ray | X |  | X |  | X |
| Methacholine challenge | X |  | X |  |  |
| 6 minute walk test | X | X (pre-dose Day 0) | X | X |  |
| Laboratory sample collection | Baseline  HIV/HBV/HCV  Hematology  Chemistry  Coagulation  Inflammatory  Urinalysis  Induced sputum | Hematology  Chemistry  Coagulation  Inflammatory  Urinalysis  Induced sputum  Research blood (Pre-Dose Day 0) | Hematology  Chemistry  Coagulation  Inflammatory  Urinalysis  Sputum  Research blood | Hematology  Chemistry  Coagulation  Inflammatory  Urinalysis  Sputum  Research blood | Hematology  Chemistry  Coagulation  Inflammatory  Urinalysis  Sputum  Research blood |
| Nasal Epithelial Cell Harvesting | X |  |  |  | X [Day 21_CO_] |
| PK Blood Sample Collection |  | Pre-dosing (Days 0, 1, 2) | X | X |  |
| Immunogenicity Blood Sample |  | X (Pre-dose Day 0) | X |  | X |
| Serum Pregnancy Tests | X |  |  |  | X [Day 21_CO_] |
| Urine Pregnancy Tests |  | X (Pre-dosing Day 0, 1, 2) |  |  |  |
| Concomitant Medications | X | X | X | X | X |
| Quality of life questionnaires |  | X (Pre-dosing Day 0) |  | X |  |

**Supplemental Table C. Study Schedule of Procedures and Evaluations**

i Vital signs include oral temperature, blood pressure, respiratory rate, pulse, and pulse oximetry.

^ii^ Day 10 spirometry performed at the discretion of study investigators.

**Supplemental Table D: List of exploratory outcomes assessed during study period.**

| Exploratory Outcomes  1. Changes in airflow before and 1 hour after administration of DAS181 or placebo as measured by the forced expiratory volume of air in 1 second (FEV1); an acute change in this measurement is defined to be a >10% change in FEV1 after inhaling drug or placebo 2. Acute changes in oxyhemoglobin saturation pre- and for 1 hour post-inhalation of study drug or placebo; a 4% decline in SpO2 will be considered significant 3. Frequency of acute exacerbations of underlying lung disease (asthma or bronchiectasis) during the active drug or placebo study period 4. Change in FEV1 from the baseline measurement (Day 0, pre-dose) in the same subject given DAS181 and given placebo at 24 hours after the third dose (Day 3). 5. Changes in daily peak expiratory flow readings for each subject during the 21 day DAS181 study period versus the 21 day placebo study period 6. Change in airway hyper-responsiveness with DAS181 versus placebo, measured as a function of the concentration of methacholine required to induce a 20% decline in FEV1 at 24 hours after last dose administered, i.e., on Day 3 7. Change in composition of sputum (viscosity and content of inflammatory cells) during each study period [Unable to analyze due to lack of consistent ability to obtain sputum] 8. Change in 6-minute walk distance and oxygen saturation with DAS181 versus placebo at 24 hours after last dose administered, i.e., on Day 3 9. Change in average reported frequency of rescue medication usage during active drug or placebo study period 10. Change in health related quality of life score, as measured by the acute Short Form-36 (SF-36) health survey and the Asthma Quality of Life Questionnaire and Asthma Control Survey between baseline and Day 10 in the active drug or placebo study period |
| --- |

**Supplemental Table E. AE grading and classification**

| - Each adverse event was graded according to the Division of AIDS Table for Grading the Severity of Adult and Pediatric Adverse Events, Version 1.0 [Updated August 2009], unless that AE was not covered by the DAIDS table. The laboratory and clinical AEs that occurred in a subject but were not specifically addressed by the DAIDS table were assessed for severity and classified into one the categories below:   - **Grade 1 (Mild):** event requires minimal or no treatment and do not interfere with the subject’s daily activities. - **Grade 2 (Moderate):** event results in a low level of inconvenience or concern with the therapeutic measures. Moderate events may cause some interference with functioning. - **Grade 3 (Severe):** event interrupts a subject’s usual daily activity and may require systemic drug therapy or other treatment. Severe events are usually incapacitating. - **Grade 4 (Life threatening):** any adverse drug experience that places the subject or participant, in the view of the investigator, at immediate risk of death from the reaction as it occurred, i.e., it does not include a reaction that had it occurred in a more severe form, might have caused death. - **Grade 5 (Death)**   - For all collected AEs, the Principle Investigator determined the adverse event’s causality based on temporal relationship and his clinical judgment. The degree of certainty about causality was graded using the categories below:   - **Definitely Related:** There is clear evidence to suggest a causal relationship, and other possible contributing factors can be ruled out. The clinical event, including an abnormal laboratory test result, occurs in a plausible time relationship to drug administration and cannot be explained by concurrent disease or other drugs or chemicals. The response to withdrawal of the drug (dechallenge) should be clinically plausible. The event must be pharmacologically or phenomenologically definitive, with use of a satisfactory rechallenge procedure if necessary. - **Probably Related:** There is evidence to suggest a causal relationship, and the influence of other factors is unlikely. The clinical event, including an abnormal laboratory test result, occurs within a reasonable time sequence to administration of the drug, is unlikely to be attributed to concurrent disease or other drugs or chemicals, and follows a clinically reasonable response on withdrawal (dechallenge). Rechallenge information is not required to fulfill this definition. - **Possibly Related:** There is some evidence to suggest a causal relationship (e.g., the event occurred within a reasonable time after administration of the trial medication). However, the influence of other factors may have contributed to the event (e.g., the subject’s clinical condition, other concomitant events). Although an adverse drug event may rate only as “possibly related” soon after discovery, it can be flagged as requiring more information and later be upgraded to “probably related” or “definitely related”, as appropriate. - **Unlikely:** A clinical event, including an abnormal laboratory test result, whose temporal relationship to drug administration makes a causal relationship improbable (e.g., the event did not occur within a reasonable time after administration of the trial medication) and in which other drugs or chemicals or underlying disease provides plausible explanations (e.g., the subject’s clinical condition, other concomitant treatments). - **Unrelated:** The AE is completely independent of study drug administration, and/or evidence exists that the event is definitely related to another cause. There must be an alternative, definitive cause documented by the clinician. - **Expected Events Related to Disease Process:** Expectedness refers to the awareness of adverse events previously observed, not on the basis of what might be anticipated from the pharmacological properties of the study agent. |
| --- |

**Supplemental Table F. List of 280 evaluable adverse events that occurred during the study period, sorted according to percent (%) frequency.**

| Adverse Event | Frequency (% of 280 total AEs) |
| --- | --- |
| Chest discomfort related to respiration | 12.5 |
| Hematuria (microscopic) | 9.64 |
| Wheezing | 8.92 |
| Dyspnea | 6.78 |
| Dry cough | 5 |
| Bacteriuria | 3.21 |
| Fatigue | 3.21 |
| Monocytosis | 3.21 |
| Nasal congestion | 3.21 |
| Elevated alkaline phosphatase | 2.86 |
| Hyperglycemia | 2.86 |
| Decreased serum albumin | 2.5 |
| Eosinophilia | 2.14 |
| Fever | 2.14 |
| Elevated blood pressure reading | 1.79 |
| Elevated C-reactive protein | 1.79 |
| Hypoglycemia | 1.79 |
| Hyponatremia | 1.79 |
| Urine white blood cell increased | 1.79 |
| Cough | 1.43 |
| Elevated Blood Urea Nitrogen (BUN) | 1.43 |
| Neutrophilia | 1.43 |
| Beta 2 microglobulin increased | 1.07 |
| Chloride increased | 1.07 |
| Elevated aspartate aminotransferase (AST) | 1.07 |
| Leukocytosis | 1.07 |
| Prolonged partial thromboplastin time (PTT) | 1.07 |
| Abnormal glomerular filtration rate | 0.71 |
| Decreased serum uric acid | 0.71 |
| Glycosuria | 0.71 |
| Hyperphosphatemia | 0.71 |
| Hypokalemia | 0.71 |
| Hypomagnesemia | 0.71 |
| Monocytopenia | 0.71 |
| Productive cough | 0.71 |
| Proteinuria | 0.71 |
| Thrombocytosis | 0.71 |
| Abdominal tenderness | 0.36 |
| Abnormal serum creatine phosphokinase (CPK) | 0.36 |
| Abnormal serum lactate dehydrogenase (LDH) | 0.36 |
| Abnormal serum protein | 0.36 |
| Acute decline in FEV1 | 0.36 |
| Anemia | 0.36 |
| Decreased hematocrit | 0.36 |
| Elevated ALT(SGPT) | 0.36 |
| FEV1 < 80% | 0.36 |
| Fibrinogen (increased) | 0.36 |
| Hypermagnesemia | 0.36 |
| Hypertension (systolic) | 0.36 |
| Hypochloremia | 0.36 |
| Hypophosphatemia | 0.36 |
| Ketonuria | 0.36 |
| Nonspecific rhinitis | 0.36 |
| Prolonged QTc | 0.36 |
